# A high-density linkage map and QTL mapping of fruit-related traits in pumpkin (*Cucurbita moschata* Duch.)

**DOI:** 10.1038/s41598-017-13216-3

**Published:** 2017-10-06

**Authors:** Yu-Juan Zhong, Yang-Yang Zhou, Jun-Xing Li, Ting Yu, Ting-Quan Wu, Jian-Ning Luo, Shao-Bo Luo, He-Xun Huang

**Affiliations:** 1 Vegetable Research Institute, Guangdong Academy of Agricultural Sciences, Guangzhou, 510640 P. R. China; 2 Guangdong Key Laboratory for New Technology Research of Vegetables, Guangzhou, 510640 P. R. China; 3 Agro-biological Gene Research Center, Guangdong Academy of Agricultural Sciences, Guangzhou, 510640 P. R. China

## Abstract

Pumpkin (*Cucurbita moschata*) is an economically worldwide crop. Few quantitative trait loci (QTLs) were reported previously due to the lack of genomic and genetic resources. In this study, a high-density linkage map of *C. moschata* was structured by double-digest restriction site-associated DNA sequencing, using 200 F2 individuals of CMO-1 × CMO-97. By filtering 74,899 SNPs, a total of 3,470 high quality SNP markers were assigned to the map spanning a total genetic distance of 3087.03 cM on 20 linkage groups (LGs) with an average genetic distance of 0.89 cM. Based on this map, both pericarp color and strip were fined mapped to a novel single locus on LG8 in the same region of 0.31 cM with phenotypic variance explained (PVE) of 93.6% and 90.2%, respectively. QTL analysis was also performed on carotenoids, sugars, tuberculate fruit, fruit diameter, thickness and chamber width with a total of 12 traits. 29 QTLs distributed in 9 LGs were detected with PVE from 9.6% to 28.6%. It was the first high-density linkage SNP map for *C. moschata* which was proved to be a valuable tool for gene or QTL mapping. This information will serve as significant basis for map-based gene cloning, draft genome assembling and molecular breeding.

## Introduction

*Cucurbita* genus is composed of five major species comprising the majority of pumpkin and squashes –*Cucurbita pepo*, *Cucurbita moschata*, *Cucurbita maxima*, *Cucurbita argyrosperma* and *Cucurbita ficifolia*. Pumpkin species, *C. moschata*, (2n = 40) is significant vegetable widely cultivated and consumed in many countries around the world^1^. Rich nutrients existing in fruit, shoot and seed are benefit for human health and offer multi-functions for pumpkin^2^. The fruit flesh is rich in carotenoids, tocopherols, polysaccharides, carbohydrates and minerals which endow pumpkin with medical functions including antidiabetic, antihypertensive, antitumor, antioxidant, immunomodulation, antibacterial, antihypercholesterolemia, intestinal antiparasitia, anti-inflammation and antalgic activities^3^. However, *C. moschata* is highly polymorphic in fruit shape, size, color and flavor phenotypes and different genotypes accumulate various levels of sugars, pigments and aroma volatiles^4^. Moreover, carotenoid and sugar traits could not be selected in a short time since the measurement is complex to achieve. Hence, it is difficult to improve the fruit quality and nutrition through traditional selective breeding based on phenotypes.

In spite of the economic and nutritional significances of *C. moschata*, there were limited genomic and genetic resources available compared with other cucurbits, such as cucumber, melon and watermelon for which microarrays^5,6^, dense genetic maps^7–9^, reverse genetic platforms^10,11^, transcriptomes^12–16^ and even whole genome sequences^17–19^ have been developed and completed. Genetic linkage map is known to be a valuable tool for high-throughput superior selection among various germplasms. In *Cucurbita* plants, a few maps for interspecific crosses or intraspecific of three species, *C. moschata*, *C. maxima* and *C. pepo* based on SSR, RAPD and AFLP markers were released^20–27^. Among them, there were only two maps for *C. moschata*: one map was composed of 205 SSR markers for studying rind color trait and the other one was composed of 95 SSR markers for detecting QTLs for chilling-stress tolerance^20,21^. However, the two maps had low density of markers and were difficult for further fine QTL mapping. With the development of next-generation sequencing technology, several reduced representation genome sequencing (RRGS) methods were developed, such as specific-locus amplified fragment sequencing (SLAF), genotyping-by-sequencing (GBS), restriction-sites associated DNA sequencing (RAD-seq) and double digest restriction-sites associated DNA sequencing (ddRAD). With these methods, a large scale SNP marker discovery is feasible and rapid to be achieved. ddRAD technology using two digesting enzymes could greatly reduce the complexity of genomes and identify abundant genetic markers quickly in an entire genome of some species with and without a reference genome, and also combines the advantages of low cost and high throughput^28^. It has been applied in tomato and peanut for high-density map construction and demonstrated to be an efficient technology^29,30^. With the reason that saturated high-density genetic map is the significant tool for QTL mapping, high-density linkage maps were constructed for cucurbits, such as cucumber, melon, watermelon, *C. maxima*, *C. pepo* and *wax gourd* ^31–37^. Taking advantage of these maps of *C. maxima* and *C. pepo*, several pumpkin QTLs were successfully identified^31,33,36^. However, there were no high density genetic maps reported for *C. moschata* which hindered QTL mapping study of this species.

Fruit morphology, flavor and nutritional metabolite trait, such as carotenoids and sugars, are important economical and nutritional traits targeted for selection in pumpkin breeding due to their contribution to yield, fruit quality and nutrition. Until recently, the unique fruit trait locus for green rind was identified for *C. moschata* which was distributed on LG5 using a map comprising 205 SSR markers^20^. Despite lack of engineering on genetic mapping of fruit-related traits in *C. moschata*, there were considerable fruit-related loci identified in other cucurbits even in *C. pepo*. In other cucurbits, fruit-related trait QTLs including fruit size, shape, color, various metabolites such as β-carotene, sugars and organic acids were identified in cucumber, melon, watermelon and wax gourd^30,34,37–44^. In *C. pepo*, fruit-related QTLs were identified including pericarp color, stripes, shape and flesh color^33^. Nevertheless, limited genes responsible for the fruit trait variation were identified such as cucumber β-carotene hydroxylase gene, cucumber *R2R3-MYB* gene and melon *Or* gene^45–47^. Therefore, with the economic and nutritional significances of fruit-related traits, especially carotenoid and sugar which have not been identified in any pumpkin species, QTL mapping is necessary to promote toward these traits in *C. moschata*.

The principal objective of the present study is to construct the first high density SNP map and identify QTLs of significant fruit-related traits in *C. moschata*. Here, we constructed a high-density linkage map with 3,470 SNP markers using ddRAD technology in a population with 200 F2 individuals composed of 20 linkage groups (LGs). Consequently, a novel gene site for both pericarp color and stripe traits was fine mapped on LG8 and a total of 29 suggestive QTLs for fruit-related traits were identified, taking advantage of the new high-density map. The genetic map and QTLs can be used for support of breeding for genetic improvement of pumpkin.

## Results

### Analysis of ddRAD data and tags development

DNA samples from two parents and 200 F2 individuals derived from CMO-1 × CMO-97 was subjected to ddRAD seq. ddRAD library was constructed using *EcoR*I and *Nla*III for digestion. By ddRAD library construction and high throughput sequencing, a total of 86.77 G data was generated with an average of 429.54 Mb for each sample (Supplementary Table S1). Among the high-quality SNPs, a total of 74,899 SNPs were polymorphic. After filtering low quality sequences, a total of 18,314 co-dominant loci SNPs which were all present in the two parents were applied for neighbor-joining tree construction (Supplementary Fig. S1). In the neighbor-joining tree, all F2 individuals were clustered normally and could be employed for downstream analysis. After discarding markers with missing rate under 20%, a total of 3,470 biallelic SNP markers were targeted for genetic map construction.

### Characteristics of the high resolution linkage genetic maps

**Figure 1:**
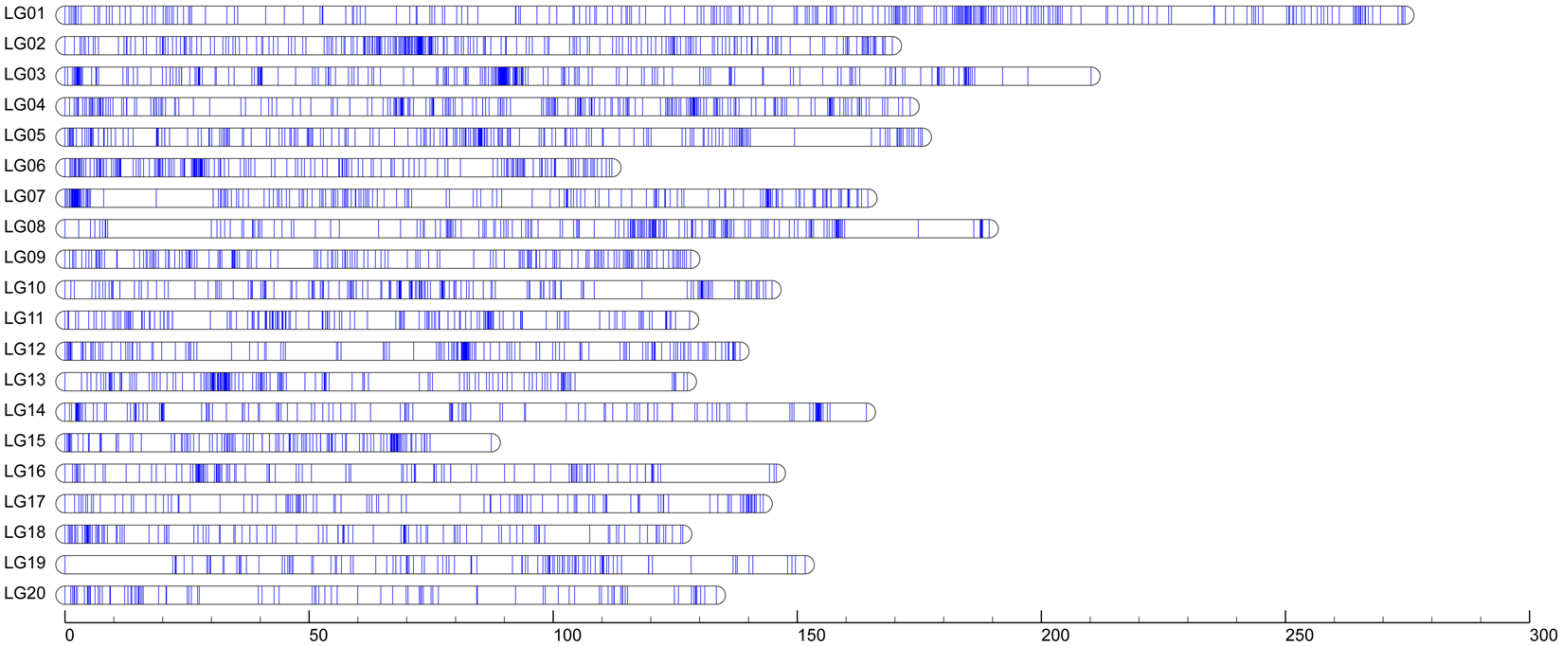
The linkage map of pumpkin (*Cucurbita moschata*) containing 20 linkage groups. A blue bar indicated a dd-RAD marker. The scaleplate on the left indicated genetic distance (centiMorgan as unit).

**Figure 2:**
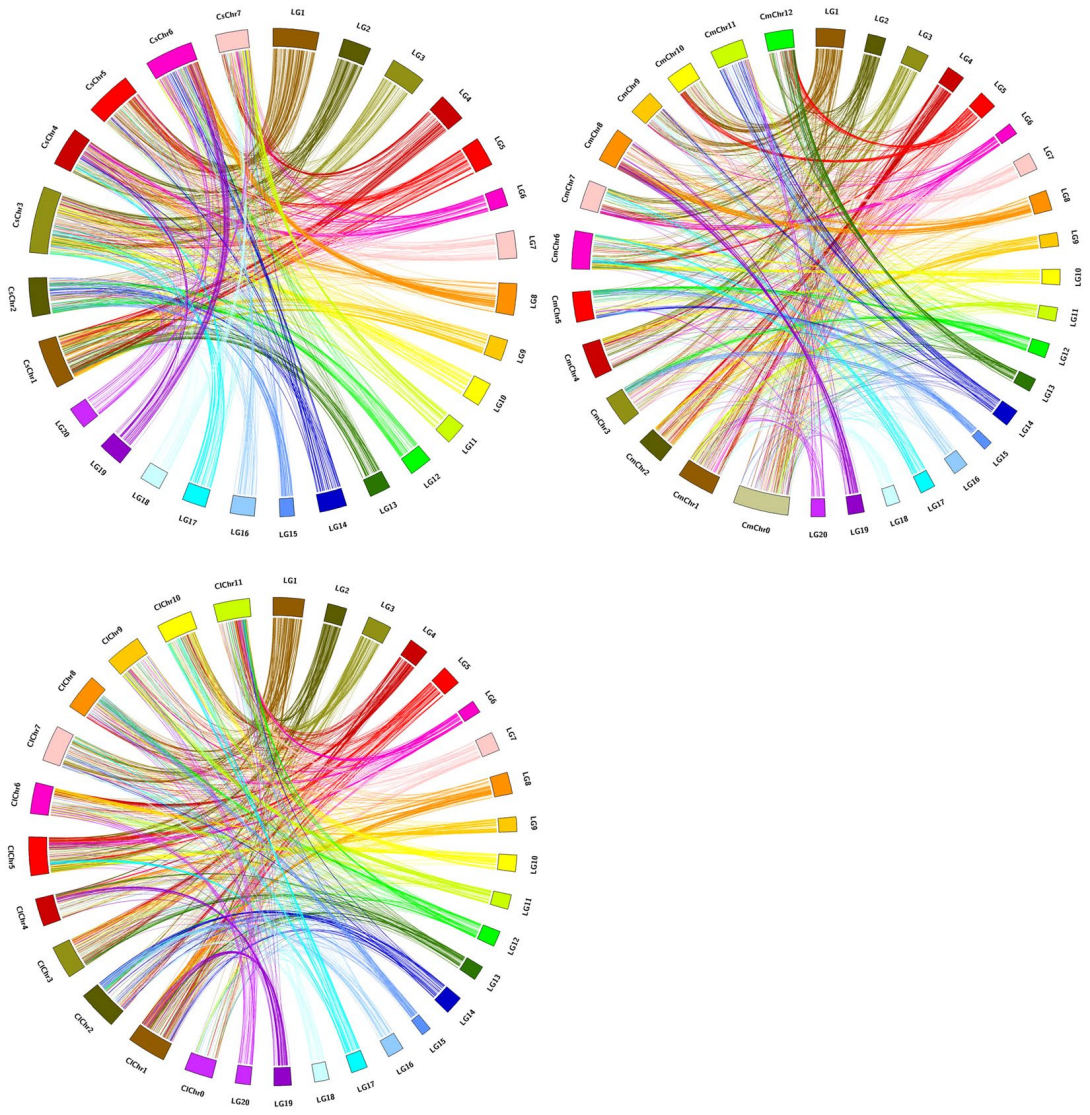
Circus mapping of 20 linkage groups of *C. moschata* to the genomic sequence of cucumber (**a**), melon (**b**) and watermelon (**c**). The pumpkin linkage groups are denoted as LGs and the pseudomolecules of cucumber, melon and watermelon are represented as CsChrs, CmChirs and ClChrs, respectively.

The 3,470 markers were mapped onto 20 LGs with a LOD (logarithm of odds threshold) of 6.0, designated LG1-LG20 using *Joinmap 4.0* software (Fig. 1). The total length of the map was determined to be 3087.03 cM with the average interval of 0.89 cM and the largest interval of 22.298 cM. About 80% of the marker intervals were within 5 cM. The sizes of LGs ranged from 87.30 cM to 247.74 cM and marker numbers in a single LG ranged from 93 to 272 (Table 1). LG1 was most saturated than other LGs with an average marker interval of 1.01 cM and the smallest LG, LG15, contained 128 markers with an average marker interval of 0.68 cM. Map length calculations were performed based on recombination frequencies. The information of all markers on the LGs was presented in Supplementary Table S2. Considering full genome sequences of *C. moschata* were not reported, all the markers in the map were involved in genome comparative mapping to genome sequences of cucumber, watermelon and melon. With sequencing identity of ≥80%, 2,073, 2,422 and 2,167 markers were selected for anchoring in cucumber, watermelon and melon genomes, respectively (Fig. 2). A part of markers on the same LGs could be anchored to the same chromosome, especially those markers in the close location.

**Table 1:** Description of basic characteristics of *Cucurbita moschata* genetic map.

Linkage	Total Marker	Total distance (cM)	Average Distance (cM)/Between marker (cM)	Largest gap(cM)	Gap(> = 5 cM)
1	272	274.47	1.01	8.70	0.12
2	264	169.47	0.64	4.22	0.00
3	249	210.18	0.84	12.93	0.22
4	247	173.10	0.70	6.39	0.07
5	213	175.62	0.82	15.74	0.18
6	201	112.03	0.55	6.65	0.06
7	199	164.47	0.83	11.66	0.25
8	188	189.31	1.01	21.24	0.29
9	173	128.17	0.74	7.42	0.11
10	166	144.81	0.87	9.77	0.21
11	159	127.95	0.80	7.75	0.16
12	158	138.25	0.87	10.48	0.24
13	159	127.48	0.80	19.91	0.28
14	139	164.15	1.18	8.86	0.32
15	128	87.30	0.68	12.51	0.21
16	119	145.70	1.22	22.29	0.31
17	117	143.01	1.22	11.02	0.22
18	114	126.54	1.10	9.20	0.20
19	112	151.62	1.35	22.09	0.39
20	93	133.41	1.43	12.09	0.41
Total	3470	3087.04	0.88	22.29	0.21

### Characterization of fruit-related traits of two parents and correlation of fruit traits

**Figure 3:**
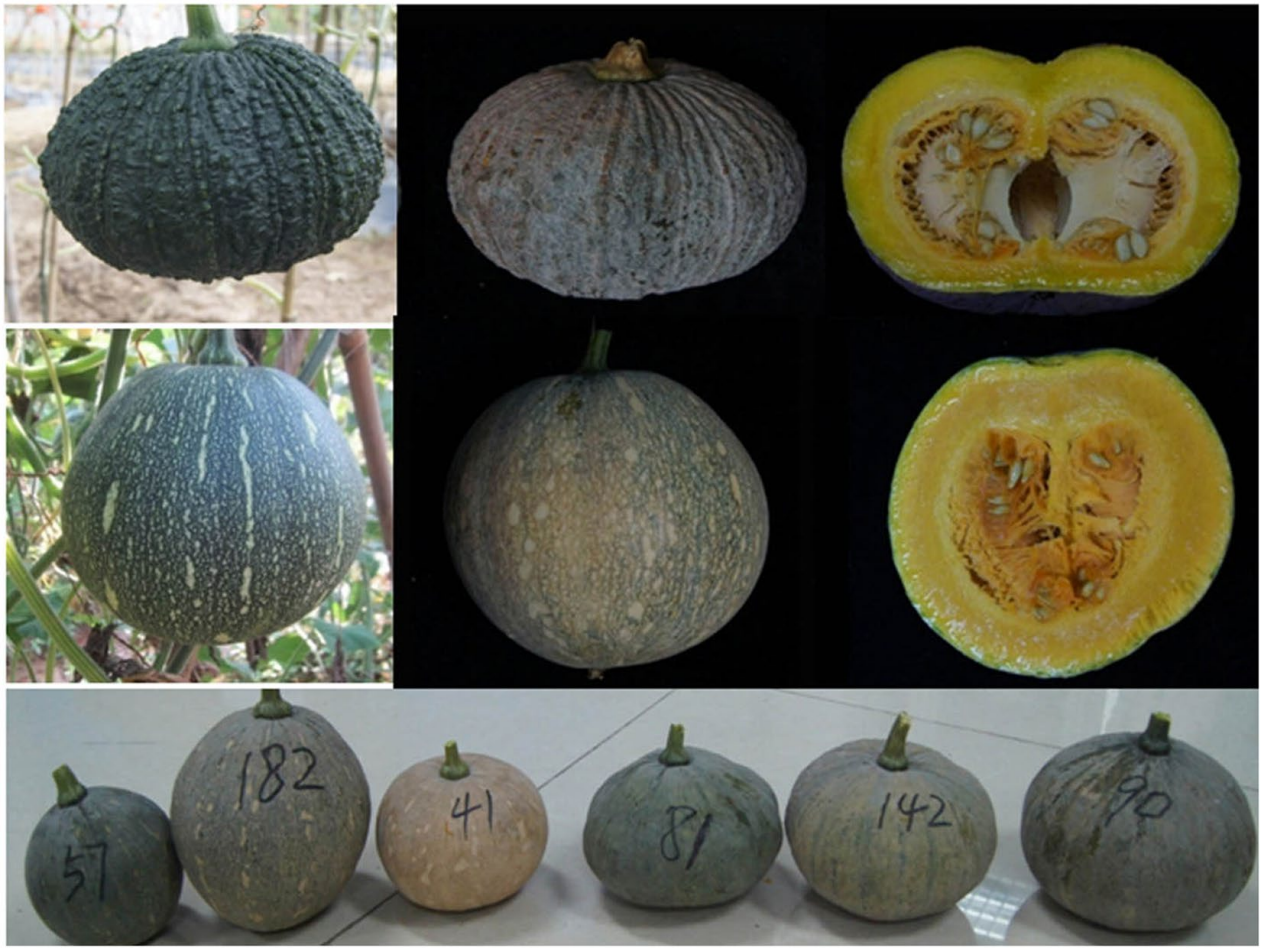
The phenotype of parental inbreds and representative F2 individuals of pumpkin. (**A**) represents CMO-1 immature fruit; (**B**) and (**C**) represent CMO-1 mature fruit; (**D**) represents CMO-97 immature fruit; (**E**) and (**F**) represent CMO-97 mature fruit; (**G**) show the phenotypes of representative F2 individuals.

Two parents, CMO-1 and CMO-97, derived from different regions, had considerable differences in fruit morphologies including pericarp color, flesh color, stripes, tuberculate, fruit shape and width (Fig. 3). The fruit flavor of two parents was also different with sweet of CMO-97 but glutinous of CMO-1. Hence, we measured fruit morphologies and quantified fruit metabolites by high performance liquid chromatography (HPLC) with a total of 14 traits (Table 2, Supplementary Fig. 2). Phenotype data (including pericarp color, stripe, lutein, β-carotene, α-carotene, total carotenoids, glucose, sucrose, glucose versus sucrose ratio, tuberculate, hollow, fruit diameter, pulp thickness and chamber width) of two parents, F1 individuals and F2 individuals were presented in Supplementary Table S2. Fruit of CMO-1 had dark green pericarp with tuberculates and no stripes while fruit of CMO-97 had light green pericarp with stripes and no tuberculates (Fig. 3). A hollow was detected in the fruit chamber of CMO-1 but not in CMO-97 (Fig. 3). Fruit diameter and chamber width were larger in CMO-1 than CMO-97 while pulp of CMO-97 was thicker than CMO-1. Carotenoid compositions were obviously different for the two parents: CMO-1 accumulated more lutein and total carotenoid contents than CMO-97 but less β-carotene and α-carotene contents than CMO-97. There were three sugars, glucose, sucrose and fructose detected in the two parents. CMO-1 accumulated higher glucose content but lower sucrose content and sucrose versus glucose ratio than CMO-97 (Table 2, Supplementary Fig. 2). There were small percentages of fructose in both parents. As a result, fructose QTL was not identified with LOD threshold of 3.0. Using person’s correlation analysis, 14 traits among the F2 population were highly correlated (Table 2), for instance, lutein was correlated with all traits except α-carotene, glucose, sucrose and tuberculate. The lowest correlation coefficients were found between β-carotene and glucose, total carotenoid and sucrose, α-carotene and pulp thickness, and glucose and tuberculate. Morphological traits were highly correlated with each other. β-Carotene, lutein, α-carotene and total carotenoid contents were highly correlated. Even though sucrose content was not correlated with glucose content, the ratio of sucrose versus glucose was correlated with each content.

**Table 2:** Pairwise correlation for all 14 fruit-related traits in *C. moschata*.

	Lutein	α-Carotene	ß-Carotene	Total c	Sucrose/glucose	Glucose	Sucrose	Pericarp color	Tuberculate fruit	Pulp thickness	Fruit diameter	Fruit hollow	Chamber width
Lutein	1.00												
α-Carotene	0.03	1.00											
ß-Carotene	0.56**	0.47**	1.00										
Total c	0.90**	0.41**	0.81**	1.00									
Sucrose/glucose	0.23*	0.28**	0.03	0.26**	1.00								
Glucose	−0.18	−0.09	−0.01	−0.16	−0.68**	1.00							
Sucrose	−0.04	0.21*	−0.07	0.01	0.38**	−0.03	1.00						
Pericarp color	−0.28**	0.00	−0.10	−0.24**	−0.12	0.12	0.08	1.00					
Tuberculate fruit	0.17	−0.12	−0.04	0.08	−0.07	0.01	−0.15	−0.28**	1.00				
Pulp thickness	0.46**	0.01	0.26**	0.42**	0.18*	−0.24**	0.08	−0.17*	0.20**	1.00			
Fruit diameter	0.40**	0.03	0.16	0.35**	0.19*	−0.22*	0.11	−0.24**	0.23**	0.76**	1.00		
Fruit hollow	−0.23*	−0.10	−0.07	−0.22*	−0.15	0.07	−0.08	0.15	−0.20*	−0.44**	−0.46**	1.00	
Chamber width	0.21*	0.03	0.03	0.17	0.13	−0.13	0.09	−0.22**	0.18*	0.34**	0.87**	−0.33**	1.00

**refers p <0.01 and * refers p <0.05.

### Fine mapping of pericarp color and pericarp stripe

**Figure 4:**
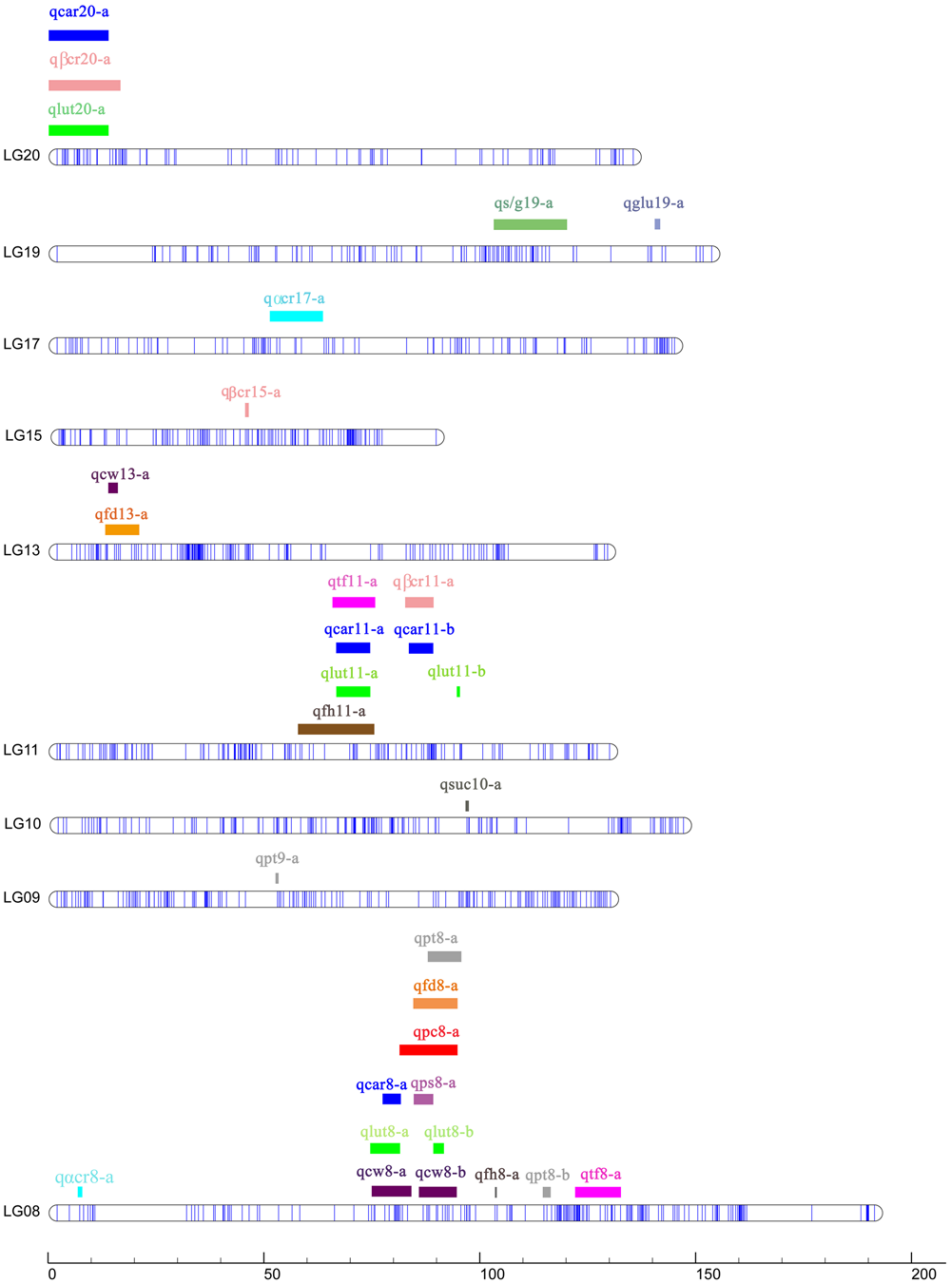
Linkage distribution of QTLs. Colored bars show the location of QTLs, and the names of QTLs are listed in Table 3.

**Figure 5:**
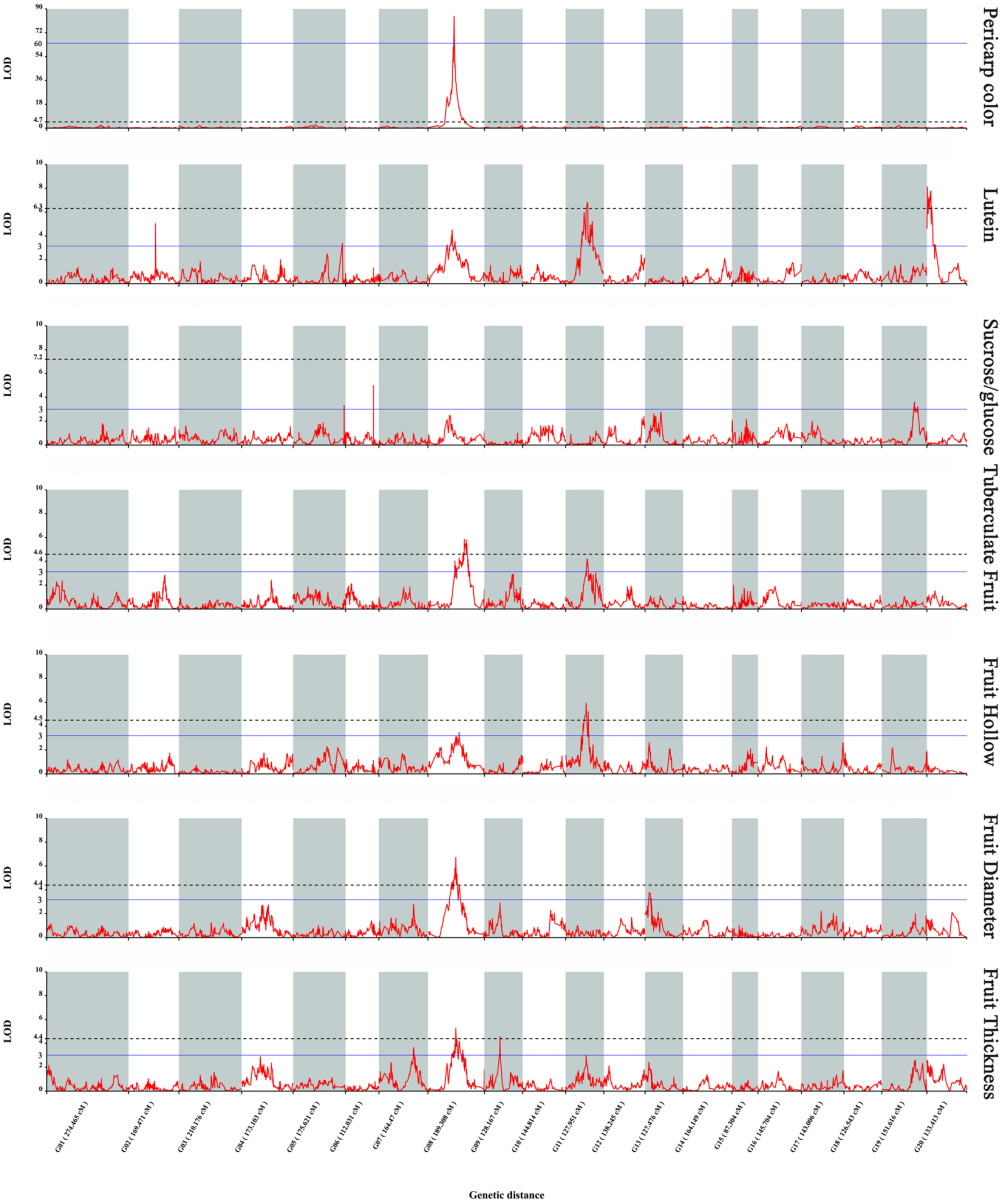
LOD scores along the 20 linkage groups for variation of the 5 traits among the 14 fruit-related. The blue horizontal line on each trait indicates the LOD = 3.0 and the imaginary line indicates the LOD for genome-wide significances for that trait.

Pericarp color of CMO-1 was dark green with no stripes in immature fruit and not easy to turn orange during maturing while the pericarp color in CMO-97 was light green with many yellow stripes and easy to turn orange (Fig. 3). The pericarp color of all the F1 individuals were light green with yellow stripes in immature fruit and turned to light orange in mature fruits. CMO-1, CMO-97, F1, F2 individuals were surveyed to study the pericarp color and stripe inheritance. Among 148 F2 individuals measured with pericarp color, the numbers of fruit with light green color and dark green pericarp color in immature fruit were 106 and 42, respectively (χ^2^ = 0.90 with P = 0.45 for 3 light green to 1 dark green). Among the 200 F2 individuals measured with pericarp stripe, the numbers of fruit with stripes and no stripes in immature fruit were 155 and 45, respectively (χ^2^ = 0.67 with P = 0.41 for 3 stripe to 1 no stripe) (Fig. 3, Table 3). It was indicated that the pericarp color and stripes were controlled by single genes, for light green dominant to dark green and stripes dominant to no stripes, which were designated as *pc* and *ps*, respectively. Based on the phenotype of F2 population and genetic map, the loci of *pc* and *ps* were mapped by MapQTL 5.0. With threshold of significance of p < 0.05 for LOD 4.7, we identified a single locus at 58.976 cM on LG8 with lowest LOD of 5.62 and highest LOD of 84.58 for *pc* (Figs 4, 5); with p < 0.05, we also identified a single locus on LG8 for *ps* that was at the same region as *ps*. On this region, marker of R1_47757 had the highest phenotypic variance explained (PVE) of 93% and 90% for *pc* and *ps*, respectively. Markers of R1_47757 and R2_63809 with a 0.31 cM region were linked to *pc* and *ps* locus.

**Table 3:** All QTLs for 14 fruit-related traits.

QTL name	Trait	LG	Position interval	LOD Threshold		LOD Max	PVE (%) Max	Add	Dom	No.of SNPs in mapped region
				Genome Threshold	Group Threshold					
qpc8-a	Pericarp color color	8	81.13–95.37	4.7	60.0	84.58	93.6	0.50	0.47	18
qps8-a	Pericarp stripe	8	84.76–89.26	4.6	50.0	71.57	90.2	0.47	0.46	8
qlut8-a	Lutein	8	73.39–81.13	6.3	3.0	4.53	15.1	−73.46	−33.83	14
qlut8-b	Lutein	8	89.26–91.63	6.3	3.0	3.58	12.2	−57.73	−38.55	5
qlut11-a	Lutein	11	68.52–74.14	6.3	6.3	6.84	22.0	−79.62	48.93	8
qlut11-b	Lutein	11	93.62–93.71	6.3	3.0	3.11	10.7	−59.27	−9.42	2
qlut20-a	Lutein	20	0–13.69	6.3	6.3	8.14	25.6	−104.90	−39.53	23
qαcr8-a	α-carotene	8	7.12–7.68	6.3	3.0	3.67	12.6	94.23	−95.49	2
qαcr17-a	α-carotene	17	50.85–64.24	6.3	3.0	5.78	19.1	28.15	−15.55	10
qβcr11-a	β-carotene	11	83.17–89.73	5.7	3.0	4.97	16.6	−26.93	12.90	20
qβcr15-a	β-carotene	15	47.07–47.58	5.7	3.0	3.02	10.5	3.24	31.84	2
qβcr20-a	β-carotene	20	0–14.61	5.7	5.7	7.45	23.8	−36.22	−19.49	25
qcar8-a	Total carotenoids	8	78.71–81.13	5.3	3.0	3.89	13.2	−92.82	−47.80	6
qcar11-a	Total carotenoids	11	68.52–74.60	5.3	3.0	5.19	17.2	−92.31	76.68	10
qcar11-b	Total carotenoids	11	84.41–89.73	5.3	3.0	4.31	14.5	−95.48	43.24	19
qcar20-a	Total carotenoids	20	0–13.69	5.3	5.3	9.29	28.6	−152.33	−56.83	23
qglu19-a	Glucose	19	140.10–140.90	4.5	3.0	3.29	11.4	−0.38	1.64	2
qsuc10-a	Sucrose	10	95.07–95.19	4.4	3.0	3.31	11.3	1.27	0.20	2
qs/g19-a	Sucrose/glucose	19	107.67–120.300	7.2	3.0	3.64	12.4	−0.14	−1.44	16
qtf8-a	Tuberculate fruit	8	120.37–132.95	4.6	4.6	5.90	16.9	−0.49	−0.12	28
qtf11-a	Tuberculate fruit	11	67.75–75.62	4.6	3.0	4.23	12.3	−0.41	0.01	13
qfh8-a	Fruit hollow	8	104.18–105.33	4.5	3.0	3.51	10.3	0.19	−0.05	5
qfh11-a	Fruit hollow	11	58.40–76.70	4.5	4.5	5.95	16.9	0.19	−0.25	19
qfd8-a	Fruit diameter	8	86.26–96.91	4.4	4.4	6.75	19.0	−1.30	−0.25	16
qfd13-a	Fruit diameter	13	13.29–20.91	4.4	3.0	3.79	11.1	−0.81	−1.01	10
qpt8-a	Pulp thickness	8	89.26–96.91	4.4	4.4	5.29	15.2	−0.30	−0.05	12
qpt8-b	Pulp thickness	8	115.92–117.27	4.4	3.0	3.48	10.3	−0.25	0.05	7
qpt9-a	Pulp thickness	9	51.04–52.41	4.4	4.4	4.60	13.4	0.01	0.44	5
qcw8-a	Chamber width	8	73.39–84.76	4.4	3.0	3.88	11.4	−0.73	0.26	15
qcw8-b	Chamber width	8	87.81–94.90	4.4	3.0	3.89	11.4	−0.70	−0.13	12
qcw13-a	Chamber width	13	14.20–17.24	4.4	3.0	3.25	9.6	−0.57	−0.49	3

PVE: phenotypic variance explained; Add: additive value; Dom: dominance value.

### QTL mapping of fruit carotenoid, sugar and shape traits

In addition to fine mapping of *pc* and *ps*, 29 QTLs for other fruit-related traits were mapped based on the phenotype data (Fig. 4., Table 3, Supplementary Fig. 3). For carotenoid traits, there were 5 QTLs for lutein content with PVE from 15.1% to 25.6%, 2 QTLs for α-carotene content with PVE from 12.6% to 19.1%, 3 QTLs for β-carotene content with PVE from 10.5% to 23.2% and 4 QTLs for total carotenoid content with PVE from 13.2% to 28.6%. For sugar traits, there was one QTL for glucose and sucrose content on LG19 and LG10 with PVE of 11.4% and 11.3%, respectively. For sucrose versus glucose ratio, one QTL on LG19 was identified in different region as glucose with PVE of 12.4%, respectively. For the fruit morphological traits, there were 2 QTLs for fruit tuberculate trait with PVE from 12.3% to 16.9%, 2 QTLs for fruit hollow with PVE from 10.3% to 16.9%, 2 QTLs for fruit diameter with PVE from 11.1% to 19.0%, 3 QTLs for fruit thickness with PVE from 10.3% to 15.2%, 3 QTLs for chamber width with PVE value from 9.6% to 11.4%. With genome LOD threshold, there were two significant QTLs for lutein and one for traits including β-carotene, total carotenoids, tuberculate, hallow, diameter and thickness among all the QTLs. Same significant QTL located on LG20 was identified for lutein, β-carotene and total carotenoids which were correlated with each other described before. Thickness and diameter were correlated in F2 population which shared the same significant QTL in the LG8. With the group threshold, the region distances for all 29 QTLs were from 0.1 cM to 18.30 cM. There were 2 to 23 markers in the QTL loci for all the QTLs. The common QTL on LG20 for lutein, β-carotene and total carotenoids had a long region of 13.69 cM which included 23 markers and could be divided into three small possible regions including 1.17cM-2.54 cM, 4.69 cM-6.152 cM and 7.12 cM-7.64 cM.

## Discussion

This study described the construction of the first high-density linkage map of *C. moschata* using SNP markers developed by ddRAD technology. Consequently, a high-density linkage map consisting of 20 LGs was constructed based on 3,470 SNPs filtered through 74,899 polymorphic SNPs. Compared with reported two genetic maps for *C. moschata* which individually comprised 205 SSR markers and 95 SSR markers, the density of this map was improved greatly^20^. Thus, for the first time, a dense linkage map of *C. moschata* was generated. Compared with other density maps of *C. maxima* and *C. pepo*, there were more individuals with 200 individuals involved in map construction^31,33^; the map length, 3087.03 cM, was longer than that reported for *C. maxima* with 2,566.8 cM and *C. pepo* with 2817.6 cM. Moreover, we identified more SNPs than *C. maxima* but lower than *C. pepo* ^31,33^. It can be explained that the variability of *C. moschata* is probably higher than *C. maxima* but lower than *C. pepo*. Based on this map, we identified a novel gene locus for pericarp color and stripe traits and 29 QTLs for other 12 fruit-related traits explaining 9.6–93.6% of the phenotypic variance. Compared with fruit-related QTL mapping of *C. pepo* using RIL population by GBS technology explaining 1.5–62.9%, the genetic map constructed in our study was sufficient for QTL mapping^33^.

In this study, *pc* and *ps* were identified to be controlled by the same single gene which was fine mapped to a region of 0.31 cM on LG8 with estimated PVE of 93.6% and 90.2%, respectively. It was previously reported that dark green with multiple mottling was dominant to light green with slight mottling in pericarp of mature fruits^20,48^. Green color locus in one population had a distance of 3.3 cM away from locus in another population and locus region were unknown as there was only a single marker in the locus^20^. In contrast, we found light green with stripes was dominant to dark green with no stripes which was predicted to be controlled by a novel gene. Based on the dense linkage map, pericarp color and stripes were fine mapped to the same small region of 0.3 cM. In zucchini, there were multiple genes controlling rind color and major QTLs controlling the rind color of the immature and mature fruit were present in different regions of LG 4 with PVE of 40.6% and 18.5%, respectively^33^. In other cucurbits, mature fruit color in cucumber was identified to be fine mapped in a single locus on chromosome 4^48^ and in wax gourd it was controlled by a single gene which was fine mapped on LG5^32^. In additon, there were 2 QTLs for stripe trait with PVE of 19.8% and 61.7% in melon^43^. Afterall, it was not consistent for genetic controlling patterns of pericarp color and stripes in different cucurbits. The *pc* and *ps* controlled by a single and novel gene mapped in this study would be used for reference for other *Cucurbita* species.

Abundance of lutein, a yellow carotenoid, in CMO-1 contributes the yellow pulp while high percentages of orange carotenoids, β-carotene and α-carotene, contribute the orange pulp of CMO-97. Due to the difference in carotenoid composition of two parents, 2 loci in LG11 and LG20 separately explained over 20% and 3 loci in LG8 and LG11 separately explained over 10% of the variation for lutein content, suggesting the high efficiency in QTL detection using ddRAD technology (Table 2, Fig. 5). There were 4 identical QTLs for lutein and total carotenoids and 2 identical QTLs for β-carotene and lutein since the three carotenoid traits were closely correlated with each other. Moreover, it was the same region controlling lutein, β-carotene and total carotenoids traits in LG20 with the PVE over 20% which suggested that QTL locus in LG20 could contain a gene regulating the carotenoid metabolic pathway in general. QTLs for minor carotenoids, α-carotene, were localized on different positions compared with other three carotenoid traits. The main reason suggested for α-carotene localized on different position as other carotenoid traits was that α-carotene was not correlated with lutein and had small correlation coefficients with other two carotenoid traits. It was reported that variation in carotenoid composition, such as, high β-carotene content, was determined by structure genes of the carotenoid biosynthetic pathway in tomato^49^. In cucumber, a locus with genetic variant in a β-carotene hydroxylase gene was identified to explain high β-carotene content^28^. Subsequently, further QTL cloning study is required to explain carotenoid traits.

Sugar is a significant quality trait of pumpkin. There are considerable different sugar composition between two parents since sucrose was the dominant sugar in CMO-97 while glucose was the dominant in CMO-1. Even though no QTLs related to Brix and fructose were detected with LOD threshold of 3.0 in present study, three single QTLs located in different locus with PVE of about 10% were identified for glucose, sucrose and ratio of glucose versus sucrose, respectively. Similarly, two single QTLs for glucose and sucrose detected were detected in watermelon using genetic linkage map constructed by CAPS and SSR markers but was in the identical locus with PVE of less than 20%^50^. In the integrated watermelon map, there were one QTL for glucose and 3 QTLs for sucrose with PVE from 6.39% to 16.71%^51^. In melon, 7 QTLs for sucrose and one for glucose were identified using RIL population with PVE from 11.3% to 19.7%^43^. It is possible that there are multiple genes controlling sucrose and glucose content in cucurbits. Nevertheless, due to no sugar QTLs cloned for cucurbits previously, more efforts on QTL cloning is necessary to reveal molecular regulation of sugar biosynthesis.

Fruit morphological traits were significant for yield and fruit quality. In this study, we identified 12 QTLs for tuberculate, hollow, diameter, thickness and chamber width traits with PVE from 9.6% to 19.0%. There were common loci for the 12 QTLs, such as in LG8 and LG13, because the fruit morphological traits were highly correlated (Table 2, Fig. 4). Interestingly, we found in LG8 which were located with several QTLs and a gene locus which could be explained that LG8 contained multiple fruit-related genes (Fig. 4). In pumpkin species, *C. pepo*, 2 QTLs for mature fruit width were identified with PVE of 7.42% to 15.14% and other fruit morphological traits, such as fruit length and shape were mapped^33^. In other cucurbits, cucumber, melon, watermelon, a number of fruit morphological QTLs have been identified^30,34,37,41,43,50,52^. However, fruit morphological candidate genes were seldom identified^33,41^. Our study firstly identified fruit morphological QTLs for *C. moschata* which paved the way for cloning candidate gene.

The developed SNP markers linked to fruit-related traits in the loci facilitates molecular breeding of new varieties through marker-assisted selection (MAS). It has been proved effective for improving quantitative and qualitative traits in cucumber^53–56^, melon^57,58^, watermelon^59,60^ even *Cucurbita* species, *C. pepo* ^61^. However, it was still in its infancy for application in *C. moschata*. Flanking markers with small mapping regions (<1 cM) have the potential to increase MAS efficacy by reducing errors during selection associated with double crossing-over. Here, the average interval of SNP markers was lower than 1 cM and there were 2-28 markers in the identified QTL regions. Thus, SNP markers in the loci were prime candidates for MAS breeding to increase gain from selection of pericarp color, stripe traits, fruit morphology traits and fruit carotenoid, sugar content traits and assisting the new variety generation. Our research will also be valuable for future genome alignment genetic improvement since genome sequences of *C. moschata* were not released. By comparing genome sequence of other cucurbits, it can provide useful information for genetic analysis prediction, linear relation and gene location speculation and evolutionary relationships. Moreover, our study will facilitate the identification of candidate genes underlying of fruit-related QTLs which can be exploited for searching new attractive market productions. The study of some traits, such as carotenoid and sugar composition, which were poorly studied, opened the new possibilities to modify the nutritional value. Subsequently, this map will allow genetic studies and molecular breeding within this species and also be elucidating for the evolution of different *Cucurbita* species.

## Materials and Methods

### Plant materials, growth condition and fruit collection

Two *C. moschata* highly inbred lines, CMO-1 and CMO-97, were employed in this study. CMO-1 was derived from Thailand and CMO-97 was derived from South China which was a ‘miben’ type germplasms. Two germplasms all bear small fruits (~1.5 kg) but exhibit contrasting phenotypes for other fruit traits, such as pericarp color, pericarp strip, flesh color and sweetness. The phenotypes and different traits of two parents were showed in detail in the results section. A population of 200 F2 individuals was generated from a single F1 plant by cross of CMO-1 × CMO-97. 20 parental lines, 20 F1 individuals and 200 F2 individuals were cultivated in the research experiment field of Vegetable Research Institute, Guangdong Academy of Agricultural Sciences, Guangzhou, China in February 2016. Germinated seeds were sown in plastic pots filled with growth medium for 2 weeks in greenhouse. Subsequently, seedlings were transplanted to field in the ridges with 30 cm in height and 200 cm in width and distance of the seedlings in rows and column was 60 cm. The ridges were filled with basal fertilizer in advance and irrigation was carried out when water was needed and fertilizer was added once before fruiting stage. Vines were leaded to climb to the trellises when it was about 2 meters long. Female flowers were pollinated with pollen from male flowers of the same plant before flowering. After one fruits sited successfully, the end flower bud in the major tendrils and lateral tendrils were cut to make sure enough nutrition for fruit. Young and healthy leaves from the two parents as well as F2 individuals were picked and frozen in liquid nitrogen immediately, and stored at − 80 °C freezer for DNA extraction which was employed for construction of high genetic density map. All self-hybridized fruits were harvested at 45 days after pollination (DAP) which were applied for fruit-related phenotypes analysis. Fruit flesh without peel and seeds were fragmented and placed in blender (Philips HR7628/00) to obtain the homogeneous mass. Fresh samples were temporary stored at −80 °C refrigerator and were then orderly freeze dried to remove water. Dried fruit samples were grounded to powder and stored permanently at −80 °C refrigerator for carotenoid and sugar measurements.

### Phenotype analysis of the parents and population

A total of 14 traits, including pericarp color, stripe, lutein, β-carotene, α-carotene, total carotenoids, glucose, sucrose, glucose versus sucrose ratio, tuberculate, hollow, fruit diameter, pulp thickness and chamber width, were analyzed. Pericarp stripe was analyzed using 10 DAP fruits of all 200 F2 individuals and pericarp color was analyzed from 20 DAP to 45 DAP fruits of 148 F2 individuals for three times through visual assessment. Other fruit shape traits were measured using fruits of 148 F2 individuals harvested at 45 DAP. Each pumpkin fruit was divided into half by longitudinal cuts at the widest cross section to measure the diameter, chamber width (chamber diameter) and pulp thickness using ruler. Hallow was visually estimated from the cross section. Tuberculate fruit was evaluated using 5 grades as follows: CMO-97 with no tuberculate was estimated as 1st grade; CMO-1 with many tuberculates in the pericarp was estimated as 5th grade; the other three type fruits with little to some tuberculates were estimated as 2st to 4st grades. Among 148 F2 individuals, fruits of 127 F2 individuals with BRIX > 4.0 representing normal accumulation of nutrient were chosen for further carotenoid and sugar measurement. Carotenoids were separated by HPLC connected with photo diode array detector (Waters, Milford, MA, USA) on a Waters Spherisorb 5 μm ODS2 4.6 mm × 250 mm column (Waters, Milford, MA, USA) as reported^62^. Carotenoids were eluted at a flow rate of 1.2 ml/min with a linear gradient from 100% solvent A [acetonitrile/methanol/0.1 M TRIS-HCl (pH 8.0), 84:2:14, v/v/v] to 100% solvent B (methanol/ethylacetate, 68:32, v/v) over a 15-min period, followed by 10 min of 100% solvent B. Carotenoids including lutein, β-carotene and α-carotene were measured at 450 nm and identified by comparing retention times and spectra against standard compounds which were finally quantified by integrating peak areas and converted them to concentrations by comparison with authentic standards that purchased from Sigma. Total carotenoid content was the sum of lutein, β-carotene and α-carotene contents. Sugars were measured using HPLC connected with a refractive index (RI) detector (Waters, Milford, MA, USA) as described by Chávez-Servín *et al*. with some modification^63^. The chromatographic separation was achieved using a Xbridge Amide (Waters, 4.6 mm × 150 mm, 3.5 μm) operating at 40 °C. The mobile phase was acetonitrile/deionized water, 8:2 (v/v) which flowed at a flow rate of 1 ml/min for 30 mins. Sugars including glucose, sucrose and fructose were quantified by comparison with authentic standards purchased from Sigma. Ratio of glucose versus sucrose was calculated in EXCEL.

### DNA extraction

Genomic DNA from two parents and their offspring was isolated by traditional phenol-chloroform extraction method in combination with RNase treatment and stored at –20 °C^64^. Before construction of ddRAD libraries, all DNA samples were quantified using a NanoDrop instrument (Thermo Scientific, DE, USA) and agarose gel electrophoresis. 202 individuals’ genomic DNA with high purity (OD_260_/_280 = _between 1.8 ~ 2.0; OD_260_/_230 = _1.8~ 2.0) and good integrity (molecular size of the primary band > 20 kb) were finally chosen for ddRAD libraries construction. Their concentrations were adjusted to 50 ng/mL using Tris-EDTA buffer.

### Library construction and sequencing

ddRAD libraries were constructed according to the method described by Peterson *et al*.^28^. Briefly, 500 ng of DNA template from each individual was double-digested using two restriction enzymes, *EcoR*I and *Nla*III (New England Biolabs [NEB], Ipswich, MA, USA; 20 U/reaction) in one combined reaction for 30 min at 37 °C. Subsequently, each fragmented sample was purified using a QiagenMinElute Reaction Cleanup Kit (Qiagen, Valencia, CA, USA) and eluted in 20 μL elution buffer (EB). Fragments were then ligated to P1 adapters (including a unique 4–8-bp multiplex identifier [MID] used to distinguish each individual) that bound to *EcoR*I-created restriction sites and P2 adapters that bound to overhangs generated by *Nla*III. In each reaction, 500 ng DNA, 1 μL P1 adapter (10 mM), 1 μL P2 adapter (10 mM), 1 μL T4 ligase (1,000 U/mL), 4 μL of 10 × T4 ligation buffer, and double-distilled water were combined into a total volume of 40 μL. The ligation was processed on a polymerase chain reaction (PCR) machine using the following conditions: 37 °C for 30 min, 65 °C for 10 min, followed by a decrease in temperature by 1.3 °C/min until the temperature reached 20 °C. Samples were pooled and size-selected (400–600 bp) from an agarose gel. Then DNA product was purified using a QiagenMinElute Gel Purication Kit and eluted in 10 μL EB. Paired-end (150 bp) sequencing of the ddRAD products from the 202 individuals was performed using an IlluminaHiSeqXten sequencing platform (Illumina, Inc., San Diego, CA, USA). Sequencing data for each individual were then extracted according to the specific MID.

### SNP discovery and genotyping

We first filtered out Illumina short reads lacking sample-specific MIDs and expected restriction enzyme motifs. Then, reads were filtered on the basis of quality score using *Trimmomatic* (v0.32)^65^ in three steps: (1) removing adapters; (2) removing reads with bases from the start or end of a read, if below the quality threshold; and (3) scanning the reads with a 4-bp sliding window, removing the read when the average Phred quality per base was below 10. The *STACKS* pipeline was used to assemble loci *de novo* from the sequencing data for SNP calling^66^. *USTACKS*, *CSTACKS*, *SSTACKS*, and *GENOTYPE* programs were used to create libraries of loci, i.e., one for each individual and one for all loci shared among individuals. The detailed parameters are as follows: USTACKS: -t gzfastq -i -m 3 -M 3 -p 15 -d -r –f –o; CSTACKS: -b 1:M 3 -p 15 -d –r; SSTACKS: -b 1 –c –p 15; GENOTYPE: -b 1 –P -r 1 -c -s -t F2. Only miss rates of less than 20% and biallelic SNPs were selected to avoid sequencing errors.

### Linkage map construction

A linkage map was constructed using *JoinMap 4.1* ^67^. Linkage group assignments were made under the logarithm of odds (LOD) score limit of 6.0. The regression mapping algorithm and Kosambi’s mapping function were used for map construction with the following settings: Rec = 0.4, LOD = 1.0, Jump = 5.

### Genome comparative mapping

To detect cross-species synteny, each amplicon or unigene was BLASTN searched against the genome sequences of cucumber^17^, watermelon^19^ and melon^18^ and the sequences were considered orthologous if sharing ≥80% sequence identity with an e-value ≤1e-5. In cases where multiplehits occurred, only the best hits were used. The software Circos was employed to visualize the genome syntenic relationships^68^.

### QTL mapping

QTLs were identified using MapQTL 5.0^69^ with multiple QTL mapping (MQM). Automatic cofactor selection (backward elimination, *P* < 0.05) was used for the detection of significantly associated markers as cofactors. LOD significance threshold levels were determined on the basis of 1,000 permutations at significance levels of *P* < 0.05. The location of each QTL was determined according to its LOD peak location and surrounding region. The percentage of the phenotypic variance explained by a QTL (R2) was estimated at the highest probability peak.

## Electronic supplementary material

Supplemental data
