# Supplementary material for: A high-density linkage map and QTL mapping of fruit-related traits in pumpkin (Cucurbita moschata Duch.)
Source: Sci Rep. 2017 Oct 6;7:12785. doi: 10.1038/s41598-017-13216-3 (PMC5630576; doi:10.1038/s41598-017-13216-3)
Supplement: Supplementary file 1 — Supplemental data [file 41598_2017_13216_MOESM1_ESM.pdf]

Supplemental information

TITLE: A high-density linkage map and QTL mapping of fruit-related traits in pumpkin (*Cucurbita moschata* Duch.)

Authors: Yu-Juan Zhong[1,2], Yang-Yang Zhou[1,2], Jun-Xing Li[1,2], Ting Yu[3], Ting-Quan Wu[1,2], Jian-Ning Luo[1], Shao-Bo Luo[1,2]*, He-Xun Huang[1]*

[1]Vegetable Research Institute, Guangdong Academy of Agricultural Sciences, Guangzhou, 510640, P. R. China

[2]Guangdong Key Laboratory for New Technology Research of Vegetables, Guangzhou, 510640,P. R. China

[3]Agro-biological Gene Research Center, Guangdong Academy of Agricultural Sciences, Guangzhou, 510640, P. R. China

* Correspondence: He-Xun Huang, huanghexun@gdaas.cn or Shao-Bo Luo, luoshaobo@gdaas.cn

Supplementary Table S1 Data generated (in Mb) per sample in parents and F2 population.

Sample name	Clean base (M)	Sample name	Clean base (M)	Sample name	Clean base (M)
CMO-97	803.75	119	528.06	230	272.66
CMO-1	641.24	121	369.86	231	354.81
1	503.98	125	585.82	233	386.16
3	397.38	126	388.31	235	342.05
4	402.36	127	293.52	236	326.72
6	407.05	132	247.23	237	273.6
7	419.09	134	358.94	239	246.77
8	341.54	136	384.4	240	245.11
9	414.71	137	307.68	243	286.93
11	323.83	138	376.65	244	269.32
12	455.77	140	338.16	246	929.41
14	439.97	141	485.27	248	973.27
15	460.16	142	722.94	249	257.68
16	443.94	143	412.69	250	497.4
19	436.59	145	421.66	251	484.24
20	365.79	146	574.42	255	494.18
25	385.84	147	331.02	256	440.3
26	337.95	150	359.84	257	549.91
27	447.07	152	327.4	258	410.61
28	365.09	153	302.46	259	518.57
30	508.5	154	307.15	260	389.65
31	369.8	155	329.05	262	490.68
32	455.21	156	387.04	263	486.11
36	1229.47	157	751.44	266	532.21
38	588.91	159	414.2	267	436.59
40	608.08	160	458.35	269	681.58
41	359.06	162	516.79	273	357.93

47	402.91	165	443.8	274	674.74
50	488.75	168	281.61	275	832.72
51	407.77	170	482.02	276	471.27
52	437.92	173	489.13	277	425.06
55	469.52	174	479.34	278	528.53
56	477.91	175	473.46	279	451.23
57	457.42	176	428.17	281	292.1
58	420.29	181	427.06	282	391.44
59	457.82	182	381.23	284	392.47
60	516.25	184	491.45	285	454.43
61	505.86	185	442.56	288	382.65
62	376.4	186	387.22	289	476.26
67	414.11	188	366.91	290	391.26
70	255.83	189	433.59	292	483.3
74	348.97	191	326.31	296	330.8
76	348.38	192	319.09	299	458.28
77	379.47	193	384.99	301	453.76
79	564.96	194	368.77	302	288.61
81	631.58	196	352.36	304	384.77
82	423.27	197	567.69	307	381.97
85	331.69	198	430.69	315	392.07
90	365.56	200	484.66	318	976.86
91	262.66	205	347.29	325	514.31
92	261.21	207	335.71	326	512.68
94	306.53	210	252.86	329	319.89
95	346.83	211	344.37	330	441.34
97	386.66	212	368.81	331	428.51
98	278.73	214	365.13	332	379.21
99	271.69	215	318.8	334	469.3
100	269.38	216	362.46	338	462.32
101	329.49	217	405.94	340	433.22
105	279.71	218	447.54	347	486.67
106	328.21	219	423.9	349	551.28
107	367.95	220	553	350	412.33
109	682.75	221	504.19	358	507.1
110	495.76	223	362.23	359	416.27
112	402.13	224	253.84	360	412.28
113	374.33	225	364.39	361	629.62
114	385.01	226	285.05	362	432.44
115	441.07	228	245.15		
117	538.56	229	243.21		

Supplementary Table S2 Fruit-related traits in parents , F1 individuals and F2 population.

Number	Stripe	Pericarp color	Lutein(μg/g)	α-Carotene (μg/g)	ß-Carotene (μg/g)	Total carotenoid(μg/g)	Glucose (μg/g)	Sucrose(μg/g)	Sucrose/glucose	Tuberculate fruit	Fruit hollow	Fruit diameter [illegible]	Pulp thickness [illegible]	Chamber width [illegible]
1	s	lg	325.62	164.55	150.39	649.21	1.69	7.48	4.43	2.00	hollow	17.20	3.00	11.20
3	ns	dg	-	-	-	-	-	-	-	3.00	solid	12.30	2.00	8.30
4	s	-	-	-	-	-	-	-	-	-	-	-	-	-
6	ns	dg	372.31	95.48	175.76	651.55	4.47	10.26	2.29	4.00	hollow	15.40	2.90	9.60
7	ns	dg	273.80	327.15	258.75	883.67	0.63	8.19	12.91	2.00	hollow	13.00	2.40	8.20
8	s	lg	286.43	102.57	115.78	513.07	2.63	8.20	3.12	2.00	hollow	15.00	2.70	9.60
9	s	lg	183.63	55.46	76.28	320.98	1.61	7.79	4.83	2.00	hollow	14.00	2.30	9.40
11	s	-	-	-	-	-	-	-	-	-	-	-	-	-
12	ns	-	-	-	-	-	-	-	-	-	-	-	-	-
14	ns	dg	736.19	85.49	243.67	1072.45	8.49	3.94	0.46	5.00	hollow	20.20	3.30	13.60
15	s	lg	-	-	-	-	-	-	-	2.00	hollow	12.40	2.00	8.40
16	s	lg	354.14	64.56	118.31	543.49	9.42	6.02	0.64	3.00	hollow	14.10	2.60	8.90
19	s	lg	376.45	111.63	190.98	686.73	7.19	6.46	0.90	4.00	hollow	13.20	1.80	9.60
20	ns	dg	-	-	-	-	-	-	-	3.00	solid	12.50	1.60	9.30
25	s	dg	363.30	82.17	124.14	578.44	2.13	5.78	2.71	3.00	hollow	17.40	3.50	10.40
26	s	lg	176.72	119.87	95.68	398.49	2.12	5.87	2.77	3.00	hollow	14.80	2.40	10.00
27	s	lg	242.91	84.48	108.43	441.78	2.41	5.49	2.28	2.00	hollow	12.60	2.60	7.40
28	ns	dg	-	-	-	-	-	-	-	4.00	hollow	12.50	2.00	8.50
30	s	lg	317.46	107.23	148.74	581.74	2.14	7.81	3.65	4.00	hollow	12.40	2.50	7.40
31	s	lg	-	-	-	-	-	-	-	2.00	hollow	13.80	2.00	9.80
32	s	lg	292.35	87.23	254.77	640.38	2.86	6.78	2.37	2.00	solid	13.00	2.60	7.80
36	ns	dg	226.61	122.05	107.35	461.72	5.74	8.06	1.40	3.00	solid	12.00	2.00	8.00
38	s	lg	211.75	131.76	73.09	427.06	7.49	5.78	0.77	2.00	hollow	12.80	2.40	8.00
40	ns	lg	360.10	52.14	89.59	510.68	4.24	7.41	1.75	3.00	hollow	15.10	3.40	8.30
41	s	lg	329.72	57.34	118.29	513.82	1.41	6.25	4.45	3.00	hollow	14.00	2.10	9.80
47	s	-	-	-	-	-	-	-	-	-	-	-	-	-

50	s	lg	317.89	107.33	156.03	589.95	1.92	9.89	5.17	4.00	hollow	12.20	2.50	7.20
51	s	lg	542.58	79.15	178.22	810.21	0.88	7.44	8.47	3.00	hollow	15.20	3.50	8.20
52	s	lg	-	-	-	-	-	-	-	2.00	solid	9.50	0.80	7.90
55	s	lg	380.74	89.64	145.64	625.98	2.68	6.06	2.26	2.00	solid	14.30	2.80	8.70
56	ns	-	-	-	-	-	-	-	-	-	-	-	-	-
57	s	dg	-	-	-	-	-	-	-	3.00	hollow	11.80	1.70	8.40
58	s	lg	360.42	202.65	186.96	759.62	7.90	9.26	1.17	3.00	hollow	15.00	3.20	8.60
59	s	lg	145.94	152.70	102.29	406.67	3.83	11.63	3.04	2.00	solid	11.90	1.90	8.10
60	s	lg	398.79	54.86	130.62	591.93	6.65	13.30	2.00	1.00	hollow	17.70	3.30	11.10
61	s	lg	-	-	-	-	-	-	-	2.00	solid	9.90	1.50	6.90
62	ns	dg	300.96	58.57	102.88	469.44	5.87	5.98	1.02	2.00	solid	12.50	2.10	8.30
67	s	lg	265.05	90.90	93.96	456.30	6.91	8.29	1.20	2.00	solid	12.20	2.30	7.60
70	ns	dg	403.72	113.68	240.79	768.60	3.87	6.95	1.80	3.00	hollow	15.30	2.80	9.70
74	s	lg	278.55	87.49	116.87	490.35	3.20	7.75	2.42	3.00	hollow	12.50	2.40	7.70
76	s	-	-	-	-	-	-	-	-	-	-	-	-	-
77	s	lg	479.00	58.01	158.80	707.59	3.79	7.33	1.94	4.00	hollow	16.00	2.20	11.60
79	s	-	-	-	-	-	-	-	-	-	-	-	-	-
81	ns	dg	287.01	54.23	100.15	447.87	4.56	4.44	0.97	4.00	hollow	16.10	3.30	9.50
82	s	lg	163.99	109.63	118.76	397.78	3.30	6.95	2.11	2.00	solid	12.50	2.00	8.50
85	ns	dg	357.89	68.06	160.34	595.46	7.96	4.17	0.52	3.00	hollow	13.30	2.70	7.90
90	s	dg	359.01	67.49	159.91	595.48	5.10	5.12	1.00	4.00	hollow	17.60	2.60	12.40
91	s	lg	179.94	87.24	95.29	367.87	2.74	7.67	2.80	2.00	hollow	17.00	2.60	11.80
92	ns	dg	468.36	65.30	113.38	659.21	2.68	6.65	2.48	4.00	hollow	16.50	2.30	11.90
94	s	lg	210.56	74.70	112.70	405.38	3.87	4.94	1.28	2.00	solid	16.00	2.80	10.40
95	s	dg	164.18	94.84	116.09	380.34	4.94	3.76	0.76	3.00	hollow	12.40	1.50	9.40
97	s	lg	246.90	71.40	181.74	505.78	3.15	0.32	0.10	3.00	solid	8.70	1.10	6.50
98	s	lg	305.44	166.35	197.19	675.93	9.64	7.35	0.76	3.00	solid	11.50	1.40	8.70
99	s	-	-	-	-	-	-	-	-	-	-	-	-	-
100	ns	-	-	-	-	-	-	-	-	-	-	-	-	-

101	s	-	-	-	-	-	-	-	-	-	-	-	-	-
105	s	lg	247.10	47.31	79.60	381.11	1.91	4.84	2.54	3.00	solid	14.00	2.40	9.20
106	s	lg	390.57	76.03	177.17	651.22	8.12	7.36	0.91	3.00	hollow	13.60	2.90	7.80
107	s	lg	375.24	122.55	131.43	638.46	2.94	6.75	2.30	4.00	hollow	13.20	2.80	7.60
109	s	-	-	-	-	-	-	-	-	-	-	-	-	-
110	s	lg	305.27	66.14	164.94	542.76	3.01	3.37	1.12	2.00	solid	12.60	2.40	7.80
112	s	lg	209.55	83.98	114.89	414.25	7.86	9.35	1.19	2.00	hollow	11.70	2.40	6.90
113	s	lg	325.86	159.87	165.93	656.64	4.41	2.19	0.50	2.00	hollow	11.10	1.70	7.70
114	s	lg	133.51	56.25	70.07	265.30	6.05	9.15	1.51	3.00	solid	10.00	1.50	7.00
115	s	-	-	-	-	-	-	-	-	-	-	-	-	-
117	s	-	-	-	-	-	-	-	-	-	-	-	-	-
119	s	lg	246.55	133.20	159.68	545.30	4.83	2.09	0.43	3.00	hollow	12.20	2.20	7.80
121	ns	dg	338.40	99.33	107.02	552.41	4.47	6.40	1.43	4.00	hollow	15.00	2.10	10.80
125	ns	dg	570.77	82.81	187.86	852.82	0.84	2.89	3.43	5.00	hollow	15.90	3.50	8.90
126	s	lg	338.86	121.74	173.32	641.75	12.01	8.33	0.69	2.00	hollow	12.60	2.20	8.20
127	s	lg	348.58	126.77	137.80	622.04	1.95	7.57	3.88	4.00	hollow	17.10	3.50	10.10
132	s	-	-	-	-	-	-	-	-	-	-	-	-	-
134	s	lg	282.09	131.43	105.33	527.01	2.46	8.38	3.40	3.00	hollow	14.80	2.20	10.40
136	s	lg	282.38	79.42	164.51	533.37	3.81	5.36	1.41	4.00	hollow	17.40	3.30	10.80
137	s	lg	428.08	111.44	191.77	740.85	6.96	5.62	0.81	3.00	hollow	15.80	2.60	10.60
138	s	-	-	-	-	-	-	-	-	-	-	-	-	-
140	ns	dg	406.25	114.38	160.52	690.48	1.85	8.80	4.75	4.00	hollow	15.20	2.10	11.00
141	ns	dg	182.67	124.38	109.62	424.52	2.09	4.43	2.12	4.00	hollow	16.40	3.20	10.00
142	ns	dg	342.22	34.07	93.28	478.28	2.06	9.17	4.46	4.00	hollow	16.80	2.80	11.20
143	s	-	-	-	-	-	-	-	-	-	-	-	-	-
145	s	lg	217.11	58.14	70.95	353.39	2.48	8.45	3.41	3.00	solid	14.60	2.00	10.60
146	ns	dg	466.01	19.74	137.41	632.86	0.72	2.15	3.01	4.00	hollow	14.30	3.30	7.70
147	s	-	-	-	-	-	-	-	-	-	-	-	-	-
150	s	lg	395.21	288.69	168.32	865.04	3.07	9.82	3.20	4.00	hollow	16.00	3.00	10.00

152	s	lg	280.41	127.47	145.55	559.95	7.03	7.86	1.12	3.00	hollow	13.50	1.80	9.90
153	s	-	-	-	-	-	-	-	-	-	-	-	-	-
154	ns	dg	346.69	171.41	131.41	658.49	0.61	3.37	5.55	3.00	hollow	13.60	2.70	8.20
155	s	lg	203.45	105.40	102.10	417.28	6.51	8.04	1.24	4.00	hollow	18.20	3.10	12.00
156	ns	-	-	-	-	-	-	-	-	-	-	-	-	-
157	s	-	-	-	-	-	-	-	-	-	-	-	-	-
159	ns	dg	262.14	37.75	129.61	433.87	3.02	0.57	0.19	3.00	solid	12.30	2.10	8.10
160	ns	-	-	-	-	-	-	-	-	-	-	-	-	-
162	s	lg	-	-	-	-	-	-	-	4.00	solid	11.70	2.00	7.70
165	s	-	-	-	-	-	-	-	-	-	-	-	-	-
168	s	lg	303.70	142.84	124.31	579.55	3.02	5.61	1.86	2.00	hollow	13.80	2.50	8.80
170	s	lg	425.07	49.64	157.45	642.16	3.83	8.01	2.09	2.00	hollow	16.90	3.90	9.10
173	s	-	-	-	-	-	-	-	-	-	-	-	-	-
174	s	lg	594.93	210.89	342.59	1159.65	4.88	4.68	0.96	2.00	hollow	16.70	3.30	10.10
175	s	dg	419.00	71.17	144.31	643.90	1.09	7.06	6.49	1.00	hollow	17.50	3.60	10.30
176	s	-	-	-	-	-	-	-	-	-	-	-	-	-
181	s	-	-	-	-	-	-	-	-	-	-	-	-	-
182	s	lg	468.93	82.98	157.12	720.33	3.53	5.10	1.44	4.00	hollow	15.30	3.10	9.10
184	s	lg	402.80	101.79	153.74	669.57	2.65	8.76	3.31	2.00	solid	11.70	2.20	7.30
185	ns	-	-	-	-	-	-	-	-	-	-	-	-	-
186	s	lg	221.65	177.31	158.38	563.87	3.18	3.31	1.04	2.00	hollow	13.90	2.40	9.10
188	s	-	-	-	-	-	-	-	-	-	-	-	-	-
189	s	lg	434.05	110.21	167.45	723.64	1.21	2.48	2.06	3.00	solid	16.90	2.80	11.30
191	s	lg	346.94	128.23	176.97	661.00	3.14	7.92	2.52	3.00	hollow	10.50	2.30	5.90
192	s	lg	142.80	150.40	91.54	390.26	0.99	6.19	6.25	2.00	hollow	14.20	2.00	10.20
193	s	lg	215.43	137.21	140.60	500.58	1.38	4.23	3.07	2.00	hollow	14.30	1.60	11.10
194	s	lg	362.92	21.68	81.51	475.20	1.35	1.63	1.20	2.00	hollow	11.00	2.50	6.00
196	ns	dg	-	-	-	-	-	-	-	4.00	hollow	11.50	2.40	6.70
197	s	lg	145.73	105.19	130.39	386.57	3.26	2.68	0.82	2.00	hollow	12.50	2.00	8.50

198	s	lg	346.32	43.64	108.85	505.19	9.69	6.93	0.72	2.00	hollow	13.30	2.20	8.90
200	s	-	-	-	-	-	-	-	-	-	-	-	-	-
205	s	lg	266.62	55.38	123.22	450.93	3.18	2.22	0.70	3.00	hollow	11.00	1.90	7.20
207	s	-	-	-	-	-	-	-	-	-	-	-	-	-
210	s	-	-	-	-	-	-	-	-	-	-	-	-	-
211	s	-	-	-	-	-	-	-	-	-	-	-	-	-
212	s	lg	406.05	50.69	157.17	620.18	5.07	1.70	0.34	4.00	solid	11.70	1.90	7.90
214	s	lg	323.17	120.76	185.94	637.42	4.15	7.59	1.83	4.00	solid	13.50	3.00	7.50
215	s	lg	485.10	159.43	143.10	798.76	0.98	6.75	6.86	4.00	hollow	17.80	3.60	10.60
216	ns	-	-	-	-	-	-	-	-	-	-	-	-	-
217	s	lg	224.10	63.55	79.85	373.05	6.47	4.30	0.66	3.00	hollow	13.00	2.00	9.00
218	s	lg	344.58	84.62	158.86	592.59	6.16	0.24	0.04	3.00	solid	10.80	1.60	7.60
219	s	lg	264.74	168.83	163.61	602.06	8.43	6.74	0.80	2.00	hollow	11.60	1.90	7.80
220	s	dg	890.20	122.73	295.18	1322.57	1.52	6.42	4.21	3.00	hollow	18.00	3.20	11.60
221	ns	-	-	-	-	-	-	-	-	-	-	-	-	-
223	s	lg	449.29	52.54	109.48	619.61	5.40	5.64	1.04	2.00	hollow	14.10	2.30	9.50
224	s	-	-	-	-	-	-	-	-	-	-	-	-	-
225	s	lg	-	-	-	-	-	-	-	2.00	hollow	8.00	1.90	4.20
226	ns	dg	207.33	112.12	125.73	449.79	7.86	5.41	0.69	3.00	hollow	15.60	2.60	10.40
228	s	lg	181.80	60.43	82.15	330.39	-	-	-	4.00	hollow	13.80	2.00	9.80
229	s	lg	126.66	87.35	94.52	313.69	6.59	12.24	1.86	2.00	hollow	13.00	1.70	9.60
230	s	lg	134.70	101.90	102.72	343.25	8.62	2.06	0.24	4.00	solid	13.10	1.80	9.50
231	ns	dg	267.23	118.99	153.63	546.85	3.40	8.58	2.53	1.00	solid	13.00	2.10	8.80
233	s	lg	93.75	57.92	62.12	218.20	7.31	5.16	0.71	2.00	solid	13.10	1.90	9.30
235	s	-	-	-	-	-	-	-	-	-	-	-	-	-
236	s	lg	213.58	36.71	84.25	341.74	3.23	8.34	2.59	2.00	hollow	12.20	1.60	9.00
237	ns	-	-	-	-	-	-	-	-	-	-	-	-	-
239	s	lg	-	-	-	-	-	-	-	3.00	solid	9.50	1.80	5.90
240	s	lg	126.66	108.21	70.72	310.42	5.26	8.05	1.53	4.00	hollow	14.20	2.70	8.80

243	s	-	-	-	-	-	-	-	-	-	-	-	-	-
244	ns	dg	478.76	143.39	134.24	769.33	0.81	8.28	10.21	4.00	hollow	14.00	2.10	9.80
246	ns	dg	421.84	80.04	87.78	598.83	1.54	5.27	3.43	4.00	hollow	17.50	3.00	11.50
248	s	-	-	-	-	-	-	-	-	-	-	-	-	-
249	s	-	-	-	-	-	-	-	-	-	-	-	-	-
250	s	lg	252.14	95.51	110.46	463.81	5.62	6.94	1.24	3.00	solid	9.00	1.80	5.40
251	ns	dg	451.76	87.33	102.99	651.61	5.97	7.94	1.33	4.00	solid	12.30	2.30	7.70
255	s	lg	238.36	112.07	163.26	518.39	7.05	6.21	0.88	3.00	hollow	12.60	2.00	8.60
256	s	-	-	-	-	-	-	-	-	-	-	-	-	-
257	s	lg	-	-	-	-	-	-	-	3.00	solid	10.30	2.00	6.30
258	ns	dg	-	-	-	-	-	-	-	4.00	hollow	13.80	2.30	9.20
259	s	lg	280.97	49.54	99.20	436.57	6.05	7.05	1.16	4.00	hollow	13.60	2.40	8.80
260	s	-	-	-	-	-	-	-	-	-	-	-	-	-
262	s	-	-	-	-	-	-	-	-	-	-	-	-	-
263	ns	dg	186.59	173.76	129.59	495.34	5.63	8.62	1.53	3.00	solid	13.90	2.10	9.70
266	s	lg	463.54	89.04	208.20	769.71	1.29	5.76	4.48	2.00	solid	11.40	2.70	6.00
267	ns	dg	288.31	27.94	102.12	425.74	6.67	2.68	0.40	3.00	hollow	17.60	3.60	10.40
269	s	lg	213.47	124.75	118.61	464.48	3.89	7.31	1.88	4.00	hollow	14.40	3.00	8.40
273	s	lg	272.34	49.80	93.97	423.49	3.54	8.56	2.42	3.00	solid	14.10	2.50	9.10
274	s	-	-	-	-	-	-	-	-	-	-	-	-	-
275	s	-	-	-	-	-	-	-	-	-	-	-	-	-
276	s	lg	284.70	50.35	107.00	449.49	6.90	5.67	0.82	3.00	solid	11.70	2.50	6.70
277	s	lg	-	-	-	-	1.89	9.78	5.18	2.00	solid	12.90	2.30	8.30
278	s	lg	-	-	-	-	-	-	-	2.00	solid	10.80	1.70	7.40
279	s	-	-	-	-	-	-	-	-	-	-	-	-	-
281	s	lg	361.91	74.89	155.99	601.93	6.21	0.46	0.07	3.00	hollow	15.20	3.10	9.00
282	s	lg	465.50	126.11	170.09	773.28	2.65	7.30	2.75	2.00	hollow	16.60	2.50	11.60
284	ns	dg	177.70	75.45	95.59	354.34	5.29	2.99	0.56	4.00	hollow	14.20	2.20	9.80
285	s	-	-	-	-	-	-	-	-	-	-	-	-	-

288	ns	dg	924.67	133.37	144.02	1218.52	1.17	7.81	6.69	2.00	hollow	13.70	2.80	8.10
289	s	dg	197.01	182.60	103.47	488.78	4.42	8.01	1.81	3.00	hollow	14.90	2.90	9.10
290	s	lg	-	-	-	-	-	-	-	1.00	solid	12.30	1.60	9.10
292	s	lg	312.67	125.62	194.92	640.97	2.86	9.26	3.23	2.00	hollow	13.80	3.00	7.80
296	s	-	-	-	-	-	-	-	-	-	-	-	-	-
299	s	lg	350.81	114.57	131.17	606.54	0.89	6.06	6.84	2.00	solid	15.00	2.00	11.00
301	s	-	-	-	-	-	-	-	-	-	-	-	-	-
302	s	lg	-	-	-	-	-	-	-	3.00	solid	9.60	1.60	6.40
304	s	lg	-	-	-	-	-	-	-	4.00	solid	11.70	1.80	8.10
307	s	lg	373.47	102.64	139.16	625.58	1.45	3.58	2.47	3.00	hollow	13.00	2.80	7.40
315	s	lg	514.27	91.81	150.15	768.62	3.25	8.73	2.69	4.00	hollow	16.60	3.20	10.20
318	s	-	-	-	-	-	-	-	-	-	-	-	-	-
325	s	-	-	-	-	-	-	-	-	-	-	-	-	-
326	s	-	-	-	-	-	-	-	-	-	-	-	-	-
329	s	lg	226.39	81.26	103.98	417.04	10.93	2.88	0.26	2.00	solid	11.00	2.10	6.80
330	s	lg	230.08	109.14	117.62	464.00	2.46	13.68	5.55	2.00	hollow	12.80	2.30	8.20
331	ns	dg	509.83	99.79	175.12	797.09	3.82	7.96	2.08	2.00	hollow	16.80	3.00	10.80
332	ns	dg	648.75	166.47	355.14	1184.62	3.87	6.16	1.59	2.00	hollow	17.10	3.70	9.70
334	s	lg	304.53	80.14	161.43	553.45	9.84	9.25	0.94	4.00	hollow	12.40	2.70	7.00
338	s	lg	394.07	67.32	123.75	592.70	11.80	4.18	0.35	3.00	hollow	13.20	2.00	9.20
340	s	lg	199.13	60.21	96.54	363.38	5.06	7.56	1.49	2.00	solid	11.50	2.00	7.50
347	s	dg	478.52	129.15	195.11	812.32	6.68	6.08	0.91	4.00	hollow	12.00	1.70	8.60
349	s	lg	357.08	89.37	173.31	625.31	6.40	2.13	0.33	3.00	solid	10.70	1.80	7.10
350	s	lg	-	-	-	-	-	-	-	3.00	hollow	13.00	2.60	7.80
358	s	-	-	-	-	-	-	-	-	-	-	-	-	-
359	s	lg	238.08	184.99	152.15	582.73	3.48	9.21	2.64	2.00	solid	12.10	1.70	8.70
360	ns	dg	273.46	68.06	106.40	454.81	3.68	5.48	1.49	4.00	hollow	15.20	3.20	8.80
361	ns	dg	-	-	-	-	-	-	-	2.00	hollow	9.90	1.50	6.90
362	s	lg	318.72	74.05	94.82	496.55	1.21	7.51	6.20	4.00	hollow	13.40	2.40	8.60

♀24	ns	dg	480.69	28.25	113.14	632.34	7.54	4.68	0.62	5.00	hollow	13.20	1.80	9.60
♀25	ns	dg	410.50	19.10	83.50	519.19	9.07	3.40	0.37	5.00	hollow	13.00	2.00	9.00
♂	s	lg	150.00	210.37	111.85	428.64	1.36	10.40	7.66	1.00	solid	9.80	2.00	5.80
♂2	s	lg	101.18	210.76	123.61	439.70	1.25	9.05	7.26	1.00	solid	12.50	2.30	7.90
F1-25	s	lg	348.92	118.90	137.42	572.32	3.98	9.35	2.35	2.00	hollow	15.50	2.40	10.70
F1-a	s	lg	222.98	87.55	111.56	485.19	5.82	8.12	1.39	4.00	hollow	14.40	2.70	9.00

Note: lg was light green ; dg was dark green ; s was stripe and ns was no stripe

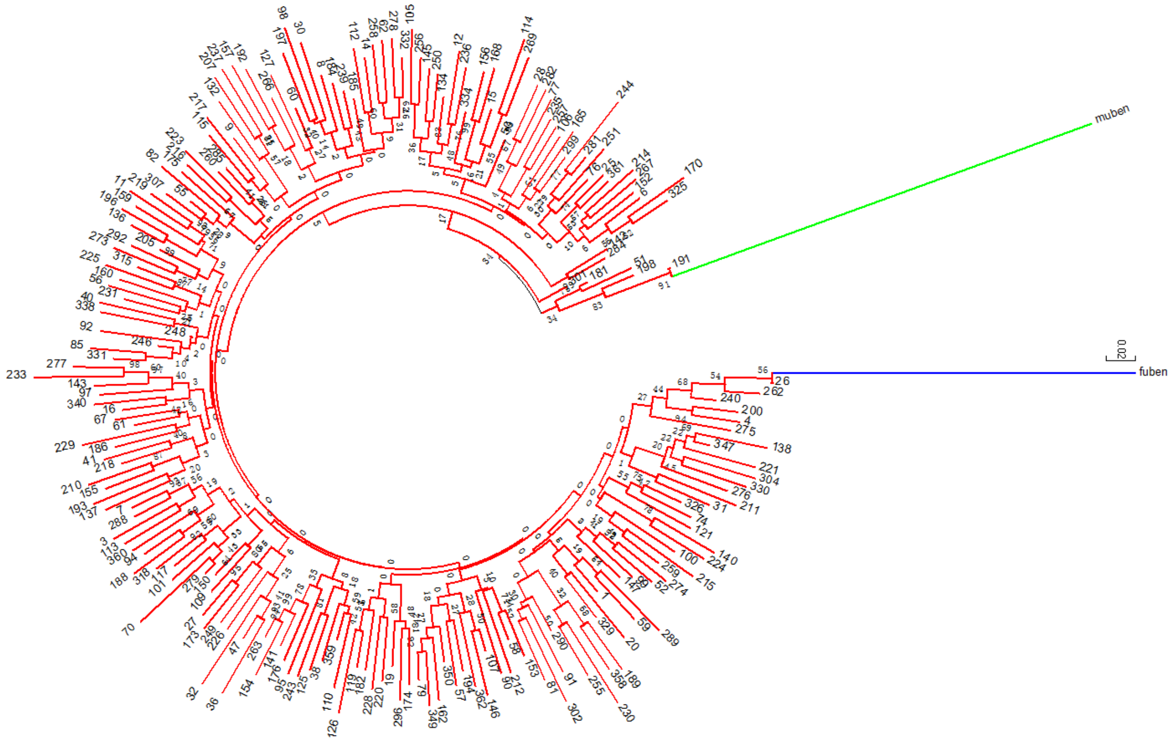

Supplementary Figure 1. Neighbor-joining tree of two parents (muben: CMO-1, fuben: CMO-97) and F2 individuals using 18,314 co-dominant loci SNPs

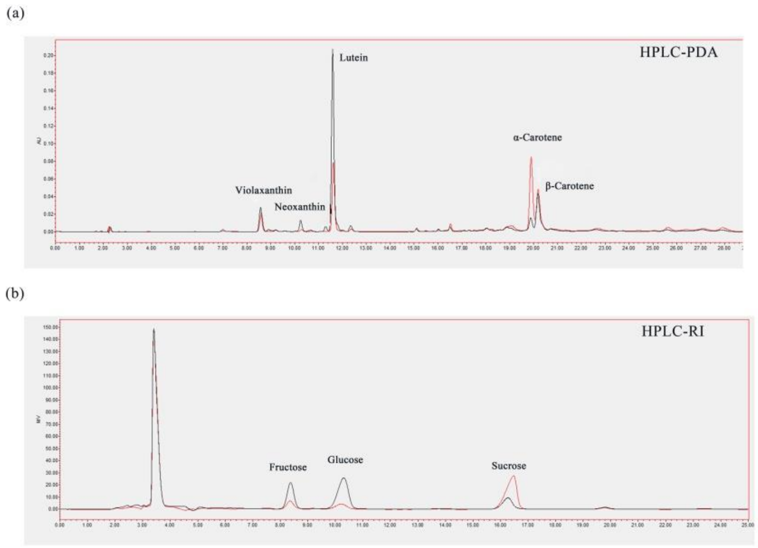

Supplementary Figure 2. HPLC elution profiles of carotenoid and sugar for parents. Red lines are represent for CMO-97 and black lines are represent for CMO-1.

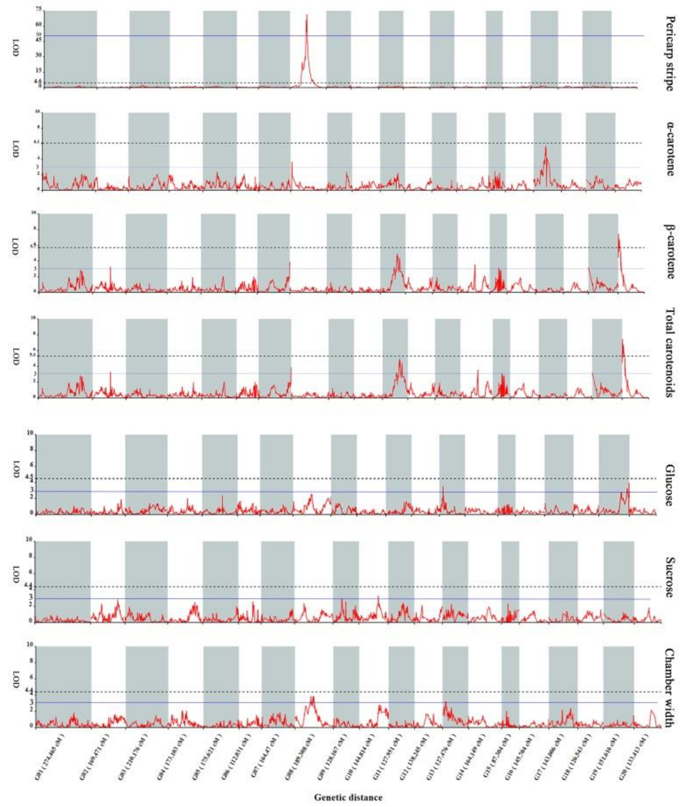

Supplementary Figure 3. Linkage distribution of QTLs for other seven fruit-related traits. Colored bars show the location of QTLs.
